# Uncommon Presentation of IgM Monoclonal Gammopathy of Undetermined Significance (MGUS) and Anti-Myelin-Associated Glycoprotein (MAG)-Associated Demyelinating Peripheral Neuropathy as Respiratory Failure: A Case Report

**DOI:** 10.7759/cureus.62865

**Published:** 2024-06-21

**Authors:** Manasawee Tanariyakul, Kensuke Takaoka, Toshiaki Takahashi, Jonathan Estaris, Kenneth Sumida

**Affiliations:** 1 Internal Medicine, John A. Burns School of Medicine, University of Hawaii, Honolulu, USA; 2 Hematology and Medical Oncology, Queen’s Cancer Center, Honolulu, USA

**Keywords:** mag-sgpg antibody, respiratory failure, cidp, mgus, anti-mag neuropathy

## Abstract

Monoclonal gammopathy of undetermined significance (MGUS) is a premalignant clonal plasma cell disorder characterized by monoclonal immunoglobulins and/or an abnormal free immunoglobulin light chain ratio. MGUS can be associated with immune-mediated neuropathies, including chronic inflammatory demyelinating neuropathy and its variants. Here, we report the case of a 76-year-old male who presented with progressive weakness, initially in the lower extremities and later including the upper extremities. Serum protein electrophoresis and immunofixation identified an IgM kappa monoclonal protein and further testing confirmed high titers of anti-myelin-associated glycoprotein (MAG) antibodies, leading to a diagnosis of anti-MAG-associated demyelinating peripheral neuropathy. The coexistence of MGUS and anti-MAG antibodies requires meticulous diagnosis and management, especially in patients who present with atypical symptoms of the disease.

## Introduction

Monoclonal gammopathy of undetermined significance (MGUS) is a premalignant clonal plasma cell disorder characterized by the presence of monoclonal immunoglobulins and/or an abnormal ratio of free immunoglobulin light chains. There are three clinical types of MGUS, including non-IgM MGUS (the most common type), IgM MGUS (accounting for 15%), and light-chain MGUS [1]. Occasionally, MGUS along with other hematologic conditions can be associated with immune-mediated neuropathies. Chronic immune-mediated neuropathies can be classified as typical chronic inflammatory demyelinating neuropathy (CIDP), CIDP variants, and other immune-mediated neuropathies not classified as CIDP. Peripheral neuropathy can be seen in up to 20% of patients with MGUS [2]. One of the CIDP variants that can be seen is distal acquired demyelinating symmetric neuropathy (DADS). DADS can be idiopathic or associated with elevated monoclonal protein (DADS-M), most often developing in the context of an IgM MGUS [3]. About 75% of DADS-M patients have anti-myelin-associated glycoprotein (MAG) antibodies [4]. There are many therapeutic options for the disease, including rituximab and cyclophosphamide that target B-cell activity [5], intravenous immunoglobulin, and plasma exchange. Currently, there is no consensus on the optimal treatment [6]. Here, we describe the case of a patient with positive anti-MAG antibodies presenting with clinical manifestations of DADS-M, including symmetric sensorimotor dysfunction of the distal extremities complicated by chronic respiratory failure.

## Case presentation

A 76-year-old male with a history of coronary artery disease and bronchiectasis presented with generalized weakness. The patient had been experiencing progressive lower extremities weakness for two years. Initially, he was found to have moderate spinal canal stenosis at L3-L4 which was treated with physical therapy. The patient then developed progressive upper extremity weakness along with numbness and tingling in both hands. The patient had also developed progressive difficulty swallowing liquids and less so solids resulting in unintentional weight loss of approximately 25 pounds or 30% of body weight in the year before admission.

Physical examination was pertinent for hypertonia of bilateral upper extremities, significant diffuse muscular atrophy in the upper and lower extremities, and muscle fasciculation seen on bilateral hands (Table 1). He had decreased pinprick, proprioception, and temperature sensation on bilateral feet and hands. Deep tendon reflex at bilateral knees, biceps, and triceps were decreased. Cerebellar signs were intact.

**Table 1:** Motor power on examination.

	Deltoid	Biceps	Wrist extension	Wrist flexion	Abductor pollicis brevis	First dorsal interosseous
Right	3	4-	5	5	5	5
Left	4	4-	5	5	5	5
	Iliopsoas	Quadriceps	Hamstrings	Ankle dorsiflexion	Ankle plantarflexion	Extensor hallucis longus
Right	5	5	5	5	5	5
Left	5	5	5	5	5	5

**Figure 1:**
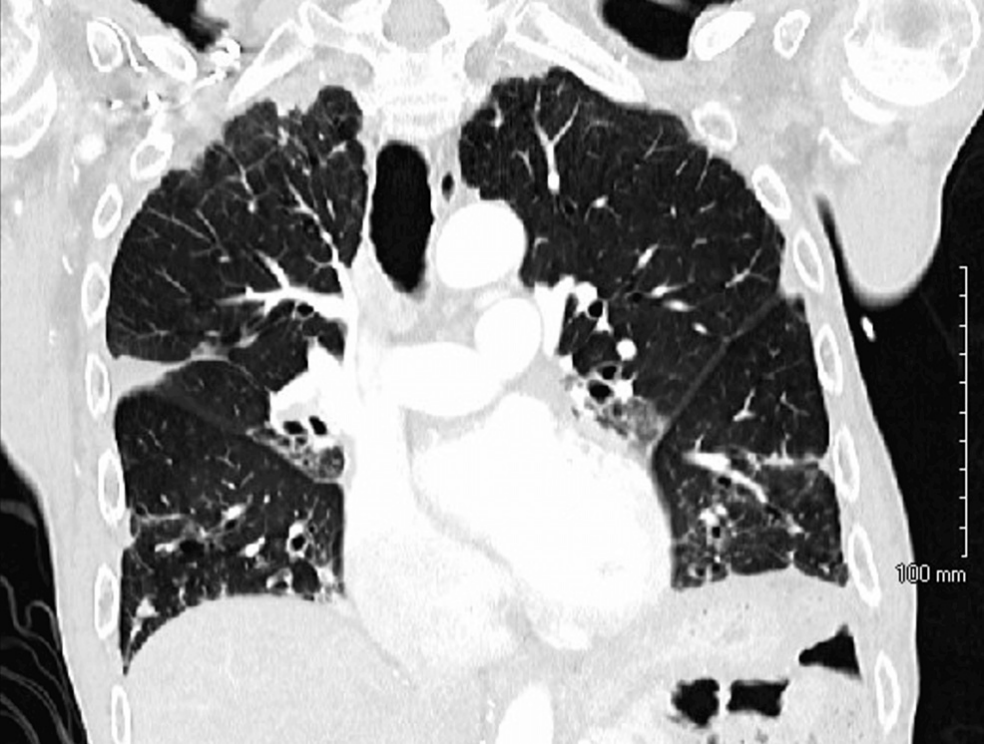
CT of the chest with contrast (coronal view) showing bronchiectasis in the lingula, right middle lobe, and lung bases.

MRI of the brain revealed mild chronic small vessel ischemic changes without other significant abnormalities. Videofluoroscopic swallow evaluation found penetration and aspiration of thin liquids as well as nectar consistency. Flexible fiberoptic laryngoscopy showed a dilated pharynx with no obstructive mass. Chest X-ray showed patchy basilar reticular opacities, worse on the right side. CT of the chest with contrast also revealed bronchial dilation and mucus plugging, suggesting bronchiectasis on the lingula, right middle lobes, and lung bases (Figure 1).

Gamma gap was elevated along with chronic respiratory acidosis with blood gas analysis revealing a pH of 7.30, partial pressure of carbon dioxide of 74 mmHg, and base excess of 10. Serum protein electrophoresis revealed a monoclonal protein in the gamma region, measuring approximately 0.40 g/dL, and serum immunofixation identified an IgM kappa monoclonal protein (Table 2). Urine protein electrophoresis did not identify obvious monoclonal proteins. An autoimmune workup was negative for anti-nuclear antibody, rheumatoid factor, anti-neutrophil cytoplasmic antibody, anti-Sjögren’s syndrome A, anti-Sjögren’s syndrome A, C3 and C4 complements, anti-double-stranded DNA, aldolase, anti-Jo-1, anti-threonyl-tRNA synthetase, anti-alanyl-tRNA synthetase, anti-glycyl-tRNA synthetase, anti-isoleucyl-transfer RNA synthetase, anti-signal recognition particle, anti-Mi-2, anti-transcription intermediary factor 1 gamma, anti-melanoma differentiation-associated gene 5, antinuclear matrix protein 2, anti-scleroderma-100, anti-U3 ribonucleoprotein, anti-U1 ribonucleoprotein, and anti-Ku. An autoimmune encephalopathy evaluation was also negative for α-amino-3-hydroxy-5-methyl-4-isoxazolepropionic acid receptor, anti-glial nuclear antibody type 1, anti-neuronal nuclear antibody type 1-3, contactin-associated protein-like 2-immunoglobulin G, dipeptidyl peptidase-like protein 6 antibody, gamma-aminobutyric acid type B receptor antibody, glutamic acid decarboxylase 65 antibody, glial fibrillary acidic protein antibody, immunoglobulin superfamily member 5, leucine-rich glioma inactivated 1-immunoglobulin G, metabotropic glutamate receptor 1 antibody, neurofilament, N-methyl-D-aspartate receptor antibody, Purkinje cell cytoplasmic antibody type 1, 2, Tr, collapsin response mediator protein 5-immunoglobulin G, neurochondrin antibody, and septin-7. An evaluation for myasthenia gravis included acetylcholine receptor antibody, anti-HMG-CoA reductase, and muscle-specific kinase autoantibody, which all returned negative. Given the concern for peripheral neuropathy in the setting of MGUS, an anti-MAG antibody assessment was done which returned positive (MAG-SGPG or myelin-associated glycoprotein sulfate-3-glucuronyl paragloboside antibody titer = 1:51,200, MAG IgM antibody titer = 1:25,600). His hospital course was complicated by worsening respiratory insufficiency resulting in hypercapnic respiratory failure requiring non-invasive ventilation. The patient declined invasive ventilatory support and advanced life support and chose comfort measures only. He passed away without efforts to treat the anti-MAG antibody-associated condition.

**Table 2:** Laboratory results during the hospitalization. Ag = antigen; Ab = antibody; MAG = myelin-associated glycoprotein; MAG-SGPG = myelin-associated glycoprotein sulfate-3-glucuronyl paragloboside

Laboratory test	Value	Reference range and units
Glucose	84	70–99 mg/dL
Blood urea nitrogen	21	6–23 mg/dL
Creatinine	0.8	0.6–1.4 mg/dL
Sodium	139	133–145 mEq/L
Potassium	4.5	3.3–5.1 mEq/L
Chloride	98	95–108 mEq/L
Carbon dioxide	36 high	21–30 mEq/L
Anion gap	10 low	14–20 mEq/L
Calcium	9.2	8.3–10.5 mg/dL
Phosphorus	3	2.5–4.5 mg/dL
Magnesium	2.1	1.6–2.6 mg/dL
Creatine kinase, total	35 low	39–308 IU/L
Aspartate aminotransferase (AST)	26	0–40 IU/L
Alanine transaminase (ALT)	12	0–41 IU/L
Alkaline phosphatase (ALP)	128 high	35–129 IU/L
Bilirubin, total	0.5	0–1.2 mg/dL
Bilirubin, direct	<0.2	0–0.3 mg/dL
Total protein	8.9 high	6.4–8.3 g/dL
Albumin	3.0 low	3.5–5.2 g/dL
Bilirubin, indirect	Not reportable	0–1.0 mg/dL
Total protein	8.9 high	6.4–8.3 g/dL
Albumin	2.68 low	3.80–5.20 g/dL
Alpha 1	0.23	0.10–0.25 g/dL
Alpha 2	0.71	0.50–1.05 g/dL
Beta	0.71	0.50–1.03 g/dL
Gamma	2.87 high	0.51–1.47 g/dL
Thyroid-stimulating hormone	2.41	0.27–4.20 µIU/mL
Ferritin	178	30–400 ng/mL
Iron, total	28 low	45–160 µg/dL
Iron binding capacity	258	228–428 µg/dL
White blood count	9.69	3.80–10.80 × 10^3^/µL
Red blood cell count	4.37	4.00–6.20 × 10^6^/µL
Hemoglobin	11.8 low	13.7–17.5 g/dL
Hematocrit	39.4 low	40.1–51.0%
Mean corpuscular volume (MCV)	90.2	79.4–98.4 fL
Mean corpuscular hemoglobin (MCH)	27	26.0–34.0 pg
Mean corpuscular hemoglobin concentration (MCHC)	29.9 low	32.0–36.0 g/dL
Red cell distribution width (RDW)	16.9 high	11.6–14.4%
Platelet count	200	151 - 424 × 10^3^/µL
Neutrophil	84.3 high	34.0–72.0%
Lymphocyte	7.1 low	12.0–44.0%
Monocyte	7.7	0.0–12.0%
Eosinophil	0.4	0.0–7.0%
Basophil	0.2	0.0–2.0%
Absolute neutrophils	8.16 high	1.56–6.20 × 10^3^/µL
Absolute lymphocytes	0.69 low	1.18–3.74 × 10^3^/µL
Absolute monocytes	0.75	0.24–0.82 × 10^3^/µL
Absolute eosinophils	0.04	0.04–0.54 × 10^3^/µL
Absolute basophils	0.02	0.01– 0.08 × 10^3^/µL
Urine protein	12	mg/dL
Urine albumin	4	mg/dL
Urine protein electrophoresis	The urine protein electrophoresis shows minimal urine protein. There is no obvious monoclonal protein identified	
Kappa free light chain urine	240.81 high	≤32.90 mg/L
Lambda free light chain urine	64.49 high	≤3.79 mg/L
Kappa/Lambda ratio	3.73	≤8.69 mg/L
Serum protein electrophoresis	The serum protein electrophoresis shows a monoclonal protein in the gamma region, measuring approximately 0.40 g/dL	
Serum immunofixation electrophoresis	IgM kappa monoclonal protein identified by immunofixation electrophoresis	
Antibodies to MAG by Western blot	Positive	Negative
Antibodies to MAG by enzyme immunoassay	1:25,600 high	Normal: <1:1,600; moderately elevated: 1:1,600–1:3,200; highly elevated: ≥1:6,400
MAG-SGPG antibody	1:51,200 high	≤1:1,600
Sedimentation rate	>130 high	0–20 mm/hour
Hepatitis B surface Ag	Negative	Negative
Hepatitis B surface Ab	Positive	Negative
Hepatitis B core Ab	Negative	Negative
Hepatitis C Ab	Negative	Negative
Immunoglobulin G (IgG)	2,550 high	700–1,600 mg/dL
Immunoglobulin A (IgA)	445 high	70–400 mg/dL
Immunoglobulin M (IgM)	889 high	40–230 mg/dL
Beta-2 microglobulin, serum	3.5 high	0.8–2.5 mg/L
Blood gas pH	7.30	7.38–7.42
Blood gas pCO_2_	74	38–42 mmHg
Blood gas base excess	10	(-2)-(+2) mmol/L

## Discussion

This case illustrates a patient with IgM MGUS who presented with at least a one-month history of symmetric bilateral sensorimotor dysfunction complicated by chronic respiratory failure and was found to have positive anti-MAG and anti-SGPG antibodies, consistent with anti-MAG-associated demyelinating peripheral neuropathy.

Anti-MAG demyelinating peripheral neuropathy represents a rare yet distinct entity within the spectrum of immune-mediated neuropathies, characterized primarily by its impact on the myelin sheath of the peripheral nervous system [7]. This condition is predominantly known for presenting with a distal, sensory-predominant polyneuropathy, leading to symptoms such as weakness, numbness, and tingling in the extremities, as noted in the present case.

To our knowledge, this case report is the second to document a patient with IgM MGUS with anti-MAG-associated demyelinating peripheral neuropathy complicated by respiratory failure. The first case report by Yuki et al. described a patient with progressive weakness of both upper extremities over two years complicated by chronic respiratory acidosis and respiratory failure [8]. The pathophysiology linking anti-MAG-associated demyelinating peripheral neuropathy with respiratory failure is not currently well understood. However, some studies suggest an association between this condition and motor neuropathy, and at least one study reported a patient with amyotrophic lateral sclerosis (ALS) and antibodies to SGPG which can cross-react with MAG [9]. Our patient also exhibited a sign of upper motor neuron involvement with increased muscle tone. Our patient passed away before confirmed nerve conduction studies and electromyography and we could not rule out the possibility of the coexistence of ALS in the patient. As upper motor neuron involvement along with chronic respiratory failure is relatively rare in anti-MAG neuropathies, this could have explained the coexistence of chronic respiratory acidosis found in our patient as well.

Our patient also had oropharyngeal dysphagia, which was one of his primary complaints. Cranial nerve involvement can be occasionally found in both typical and atypical CIDP. His bronchiectasis prominent on the basal lungs was likely contributing to his dysphagia as well. The frequency of cranial nerve palsy was 11% in typical CIDP and 11% in DADS [10]. A case report by Yoshida et al. reported a patient with IgM monoclonal gammopathy with anti-MAG and SGPG antibodies presenting with cranial neuropathy, including unilateral facial palsy, dysesthesia, dysarthria, and dysphagia [11]. Furthermore, a case report by Drouet et al. presented a patient with IgM monoclonal gammopathy with progressive distal sensorimotor neuropathy along with cranial neuropathy involving the oro-pharyngo-laryngo territory [12]. Although cranial neuropathy in anti-MAG-associated demyelinating peripheral neuropathy is rare, these case reports reiterate the rare manifestations of patients with such a disease.

## Conclusions

This case report contributes to the limited but growing evidence on the association of anti-MAG-associated demyelinating peripheral neuropathy with chronic respiratory failure. This complexity also underscores the necessity to consider anti-MAG antibodies and monoclonal gammopathy in patients who present with demyelinating peripheral neuropathy.
